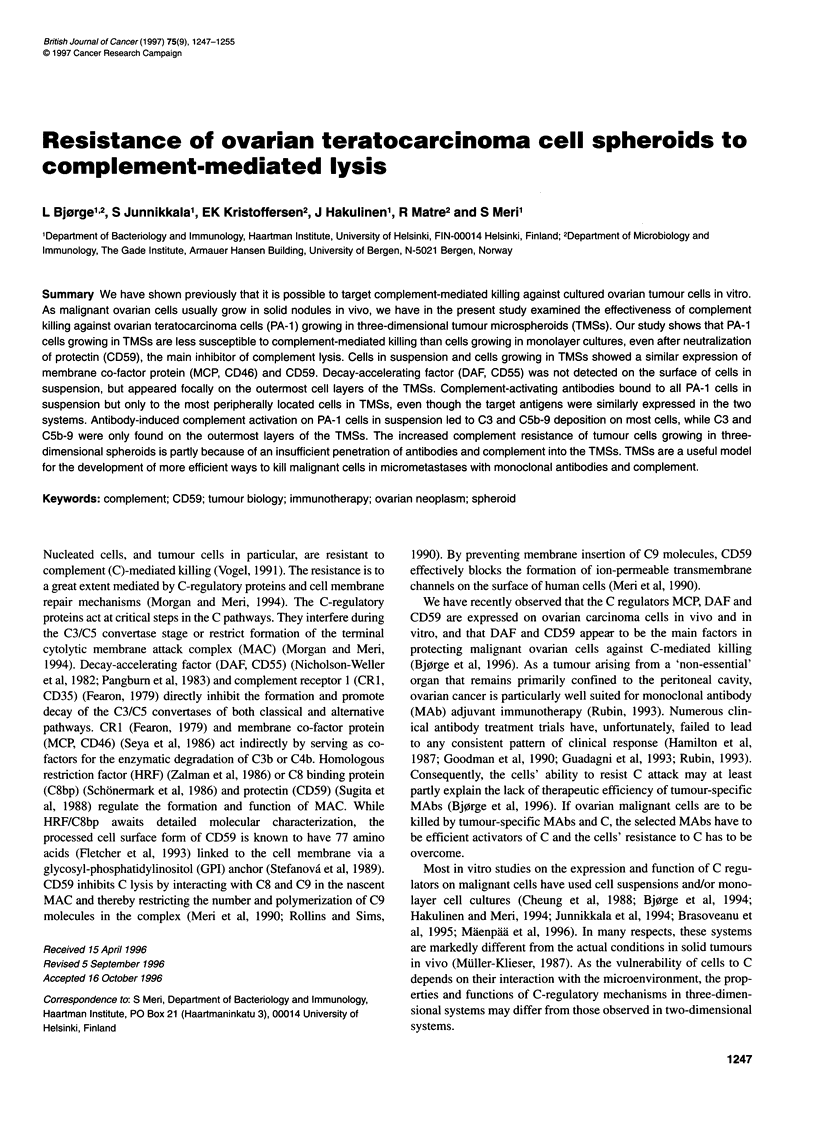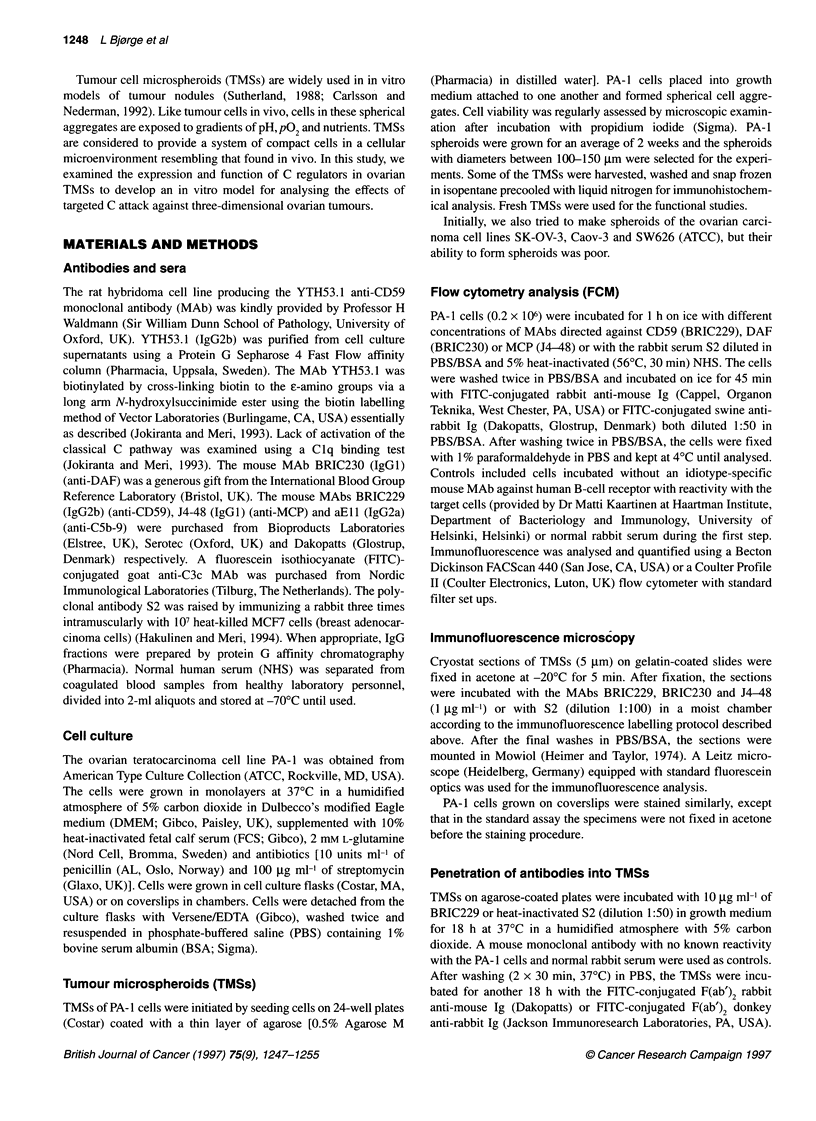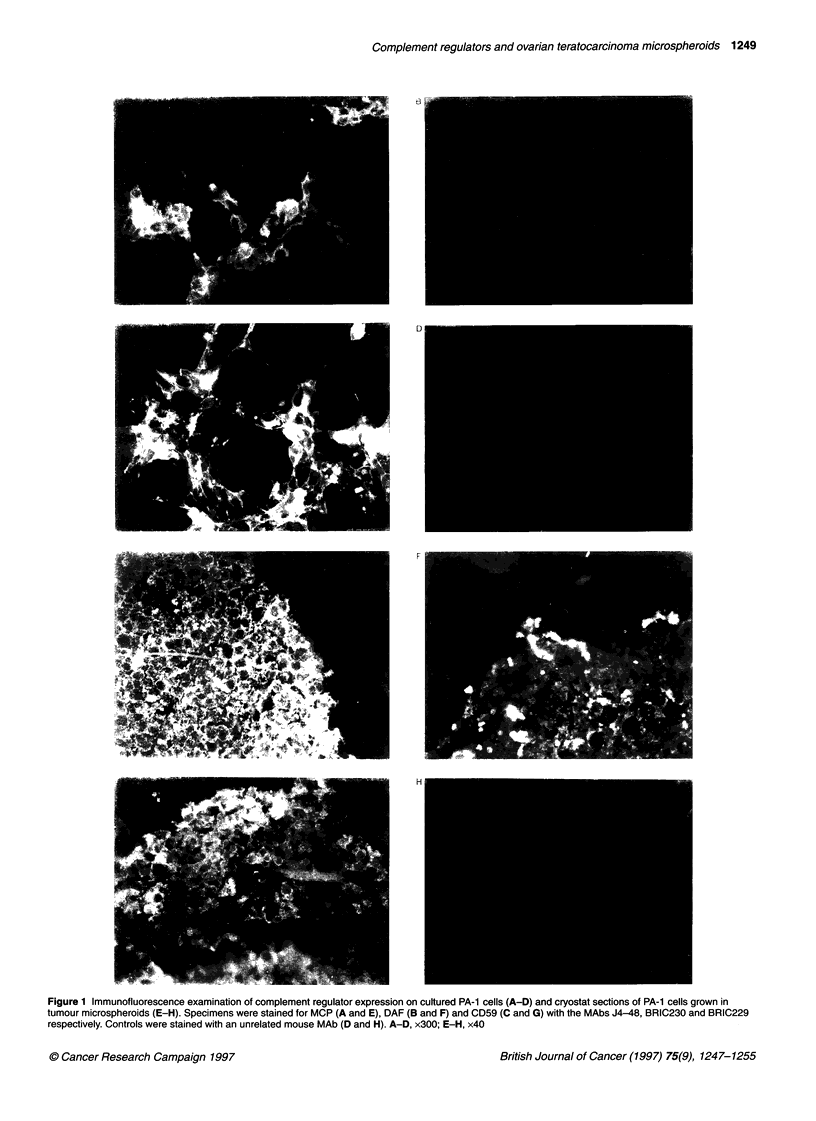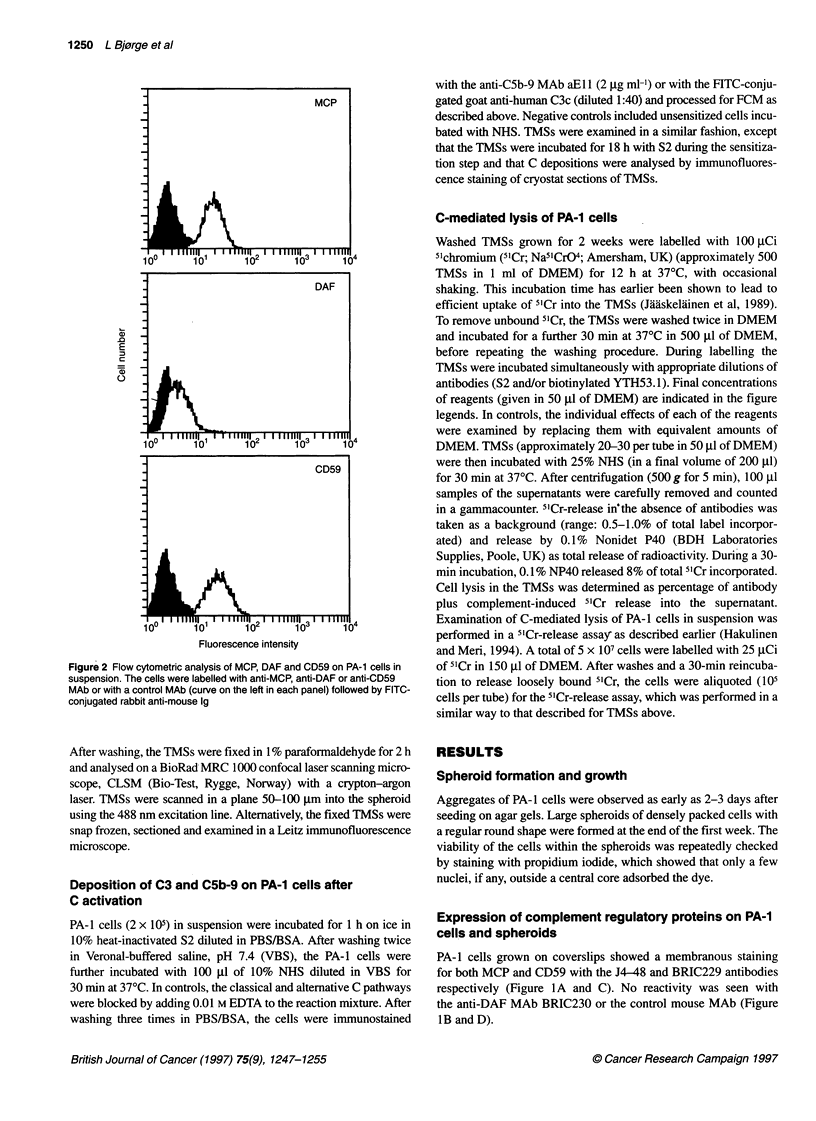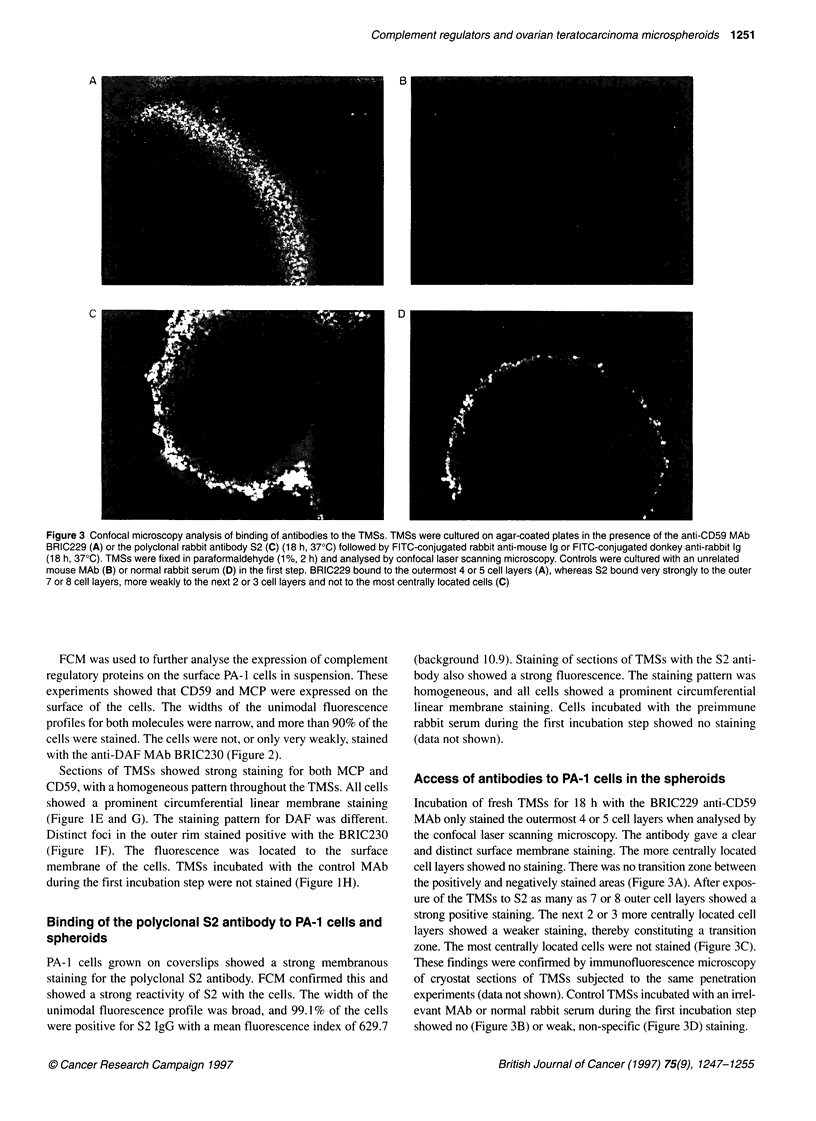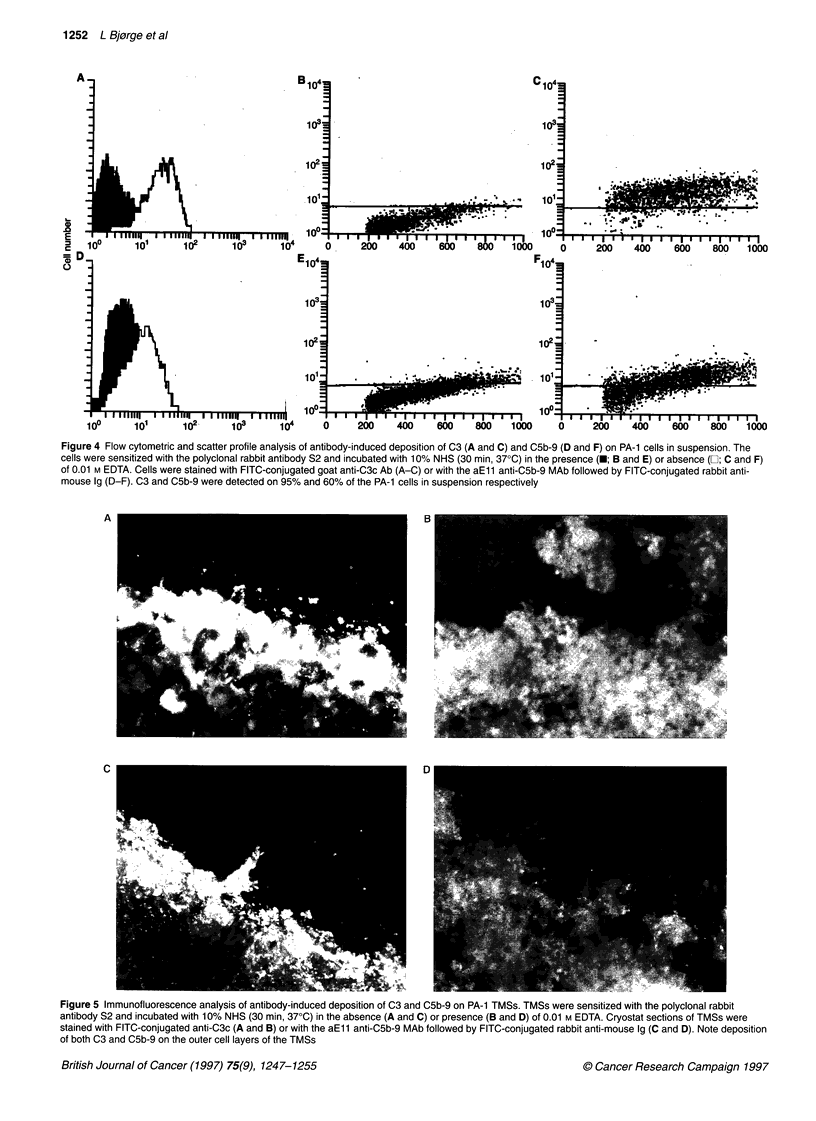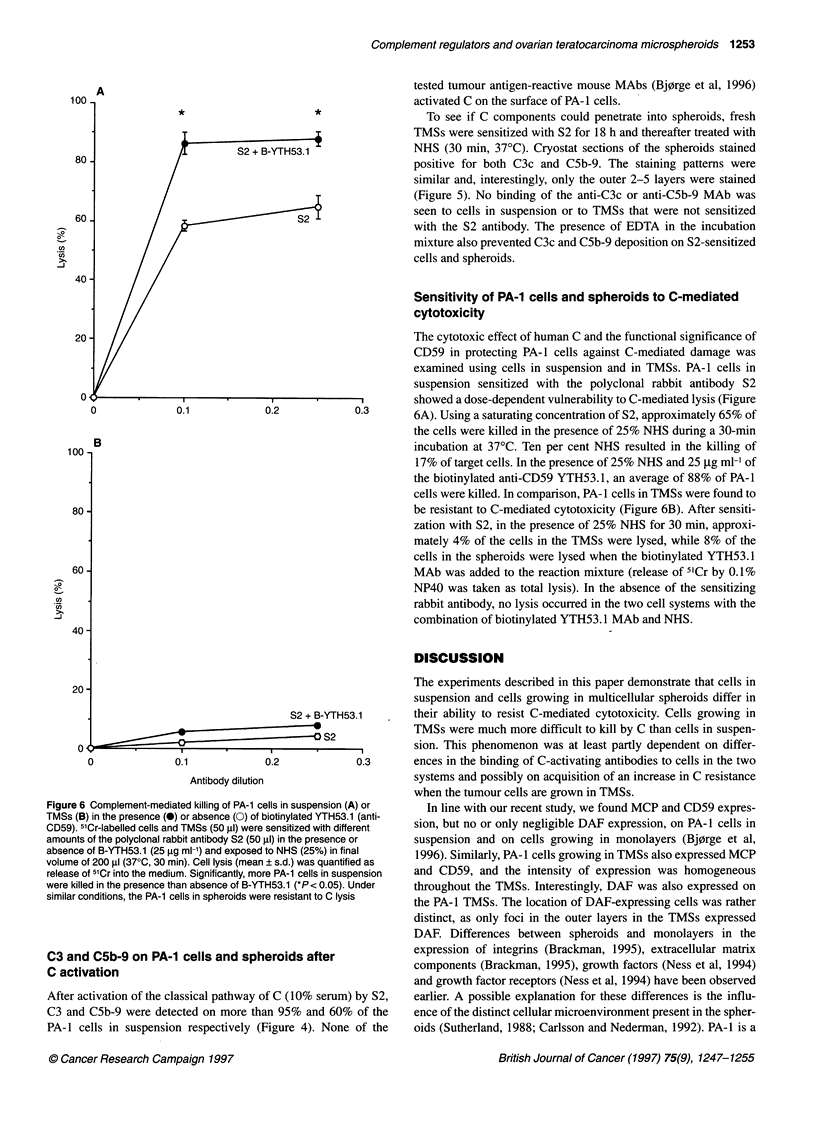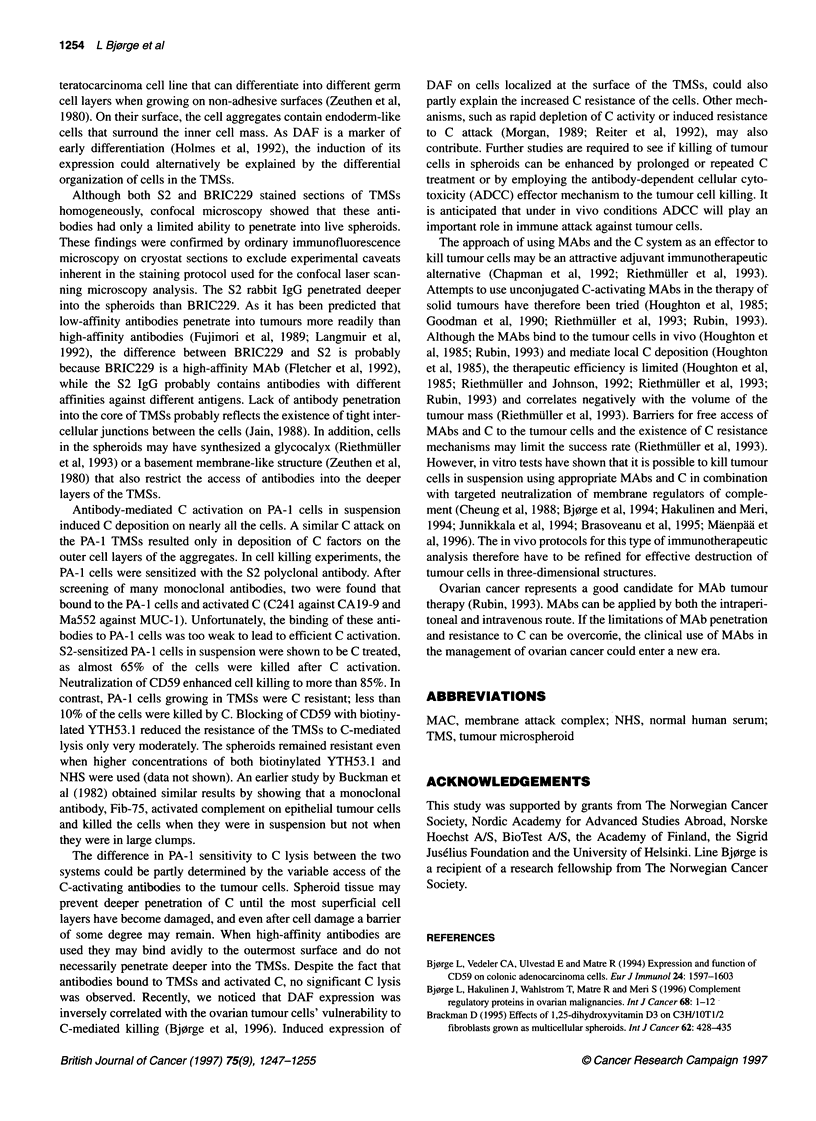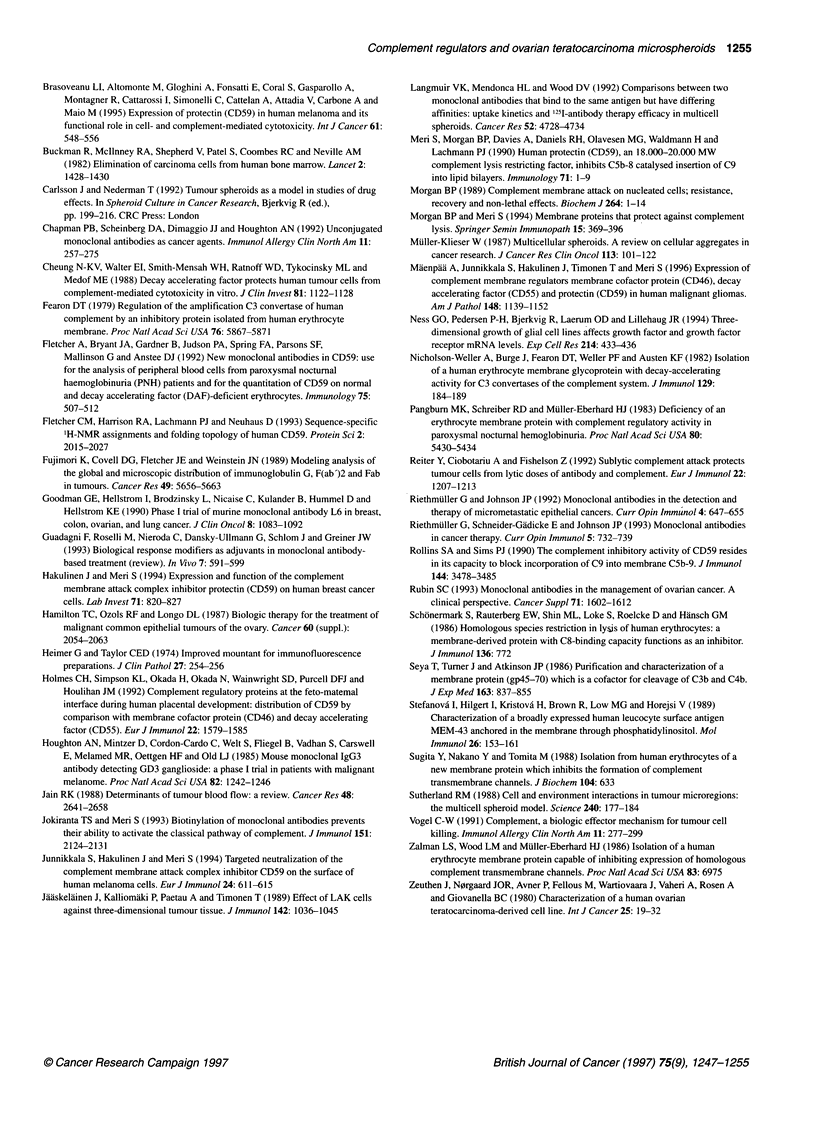# Resistance of ovarian teratocarcinoma cell spheroids to complement-mediated lysis.

**DOI:** 10.1038/bjc.1997.213

**Published:** 1997

**Authors:** L. BjÃ¸rge, S. Junnikkala, E. K. Kristoffersen, J. Hakulinen, R. Matre, S. Meri

**Affiliations:** Department of Bacteriology and Immunology, Haartman Institute, University of Helsinki, Finland.

## Abstract

**Images:**


British Journal of Cancer (1997) 75(9), 1247–1255
© 1997 Cancer Research Campaign

### Resistance of ovarian teratocarcinoma cell spheroids to complement-mediated lysis

**L Bjørge[1,2], S Junnikkala[1], EK Kristoffersen[2], J Hakulinen[1], R Matre[2] and S Meri[1]**

[1]Department of Bacteriology and Immunology, Haartman Institute, University of Helsinki, FIN-00014 Helsinki, Finland; [2]Department of Microbiology and Immunology, The Gade Institute, Armauer Hansen Building, University of Bergen, N-5021 Bergen, Norway

**Summary** We have shown previously that it is possible to target complement-mediated killing against cultured ovarian tumour cells in vitro. As malignant ovarian cells usually grow in solid nodules in vivo, we have in the present study examined the effectiveness of complement killing against ovarian teratocarcinoma cells (PA-1) growing in three-dimensional tumour microspheroids (TMSs). Our study shows that PA-1 cells growing in TMSs are less susceptible to complement-mediated killing than cells growing in monolayer cultures, even after neutralization of protectin (CD59), the main inhibitor of complement lysis. Cells in suspension and cells growing in TMSs showed a similar expression of membrane co-factor protein (MCP, CD46) and CD59. Decay-accelerating factor (DAF, CD55) was not detected on the surface of cells in suspension, but appeared focally on the outermost cell layers of the TMSs. Complement-activating antibodies bound to all PA-1 cells in suspension but only to the most peripherally located cells in TMSs, even though the target antigens were similarly expressed in the two systems. Antibody-induced complement activation on PA-1 cells in suspension led to C3 and C5b-9 deposition on most cells, while C3 and C5b-9 were only found on the outermost layers of the TMSs. The increased complement resistance of tumour cells growing in three-dimensional spheroids is partly because of an insufficient penetration of antibodies and complement into the TMSs. TMSs are a useful model for the development of more efficient ways to kill malignant cells in micrometastases with monoclonal antibodies and complement.

**Keywords:** complement; CD59; tumour biology; immunotherapy; ovarian neoplasm; spheroid

Nucleated cells, and tumour cells in particular, are resistant to complement (C)-mediated killing (Vogel, 1991). The resistance is to a great extent mediated by C-regulatory proteins and cell membrane repair mechanisms (Morgan and Meri, 1994). The C-regulatory proteins act at critical steps in the C pathways. They interfere during the C3/C5 convertase stage or restrict formation of the terminal cytolytic membrane attack complex (MAC) (Morgan and Meri, 1994). Decay-accelerating factor (DAF, CD55) (Nicholson-Weller et al, 1982; Pangburn et al, 1983) and complement receptor 1 (CR1, CD35) (Fearon, 1979) directly inhibit the formation and promote decay of the C3/C5 convertases of both classical and alternative pathways. CR1 (Fearon, 1979) and membrane co-factor protein (MCP, CD46) (Seya et al, 1986) act indirectly by serving as co-factors for the enzymatic degradation of C3b or C4b. Homologous restriction factor (HRF) (Zalman et al, 1986) or C8 binding protein (C8bp) (Schönermark et al, 1986) and protectin (CD59) (Sugita et al, 1988) regulate the formation and function of MAC. While HRF/C8bp awaits detailed molecular characterization, the processed cell surface form of CD59 is known to have 77 amino acids (Fletcher et al, 1993) linked to the cell membrane via a glycosyl-phosphatidylinositol (GPI) anchor (Stefanová et al, 1989). CD59 inhibits C lysis by interacting with C8 and C9 in the nascent MAC and thereby restricting the number and polymerization of C9 molecules in the complex (Meri et al, 1990; Rollins and Sims, 1990). By preventing membrane insertion of C9 molecules, CD59 effectively blocks the formation of ion-permeable transmembrane channels on the surface of human cells (Meri et al, 1990).

*Received 15 April 1996*
*Revised 5 September 1996*
*Accepted 16 October 1996*

*Correspondence to*: S Meri, Department of Bacteriology and Immunology, Haartman Institute, PO Box 21 (Haartmaninkatu 3), 00014 University of Helsinki, Finland

We have recently observed that the C regulators MCP, DAF and CD59 are expressed on ovarian carcinoma cells in vivo and in vitro, and that DAF and CD59 appear to be the main factors in protecting malignant ovarian cells against C-mediated killing (Bjørge et al, 1996). As a tumour arising from a 'non-essential' organ that remains primarily confined to the peritoneal cavity, ovarian cancer is particularly well suited for monoclonal antibody (MAb) adjuvant immunotherapy (Rubin, 1993). Numerous clinical antibody treatment trials have, unfortunately, failed to lead to any consistent pattern of clinical response (Hamilton et al, 1987; Goodman et al, 1990; Guadagni et al, 1993; Rubin, 1993). Consequently, the cells' ability to resist C attack may at least partly explain the lack of therapeutic efficiency of tumour-specific MAbs (Bjørge et al, 1996). If ovarian malignant cells are to be killed by tumour-specific MAbs and C, the selected MAbs have to be efficient activators of C and the cells' resistance to C has to be overcome.

Most in vitro studies on the expression and function of C regulators on malignant cells have used cell suspensions and/or monolayer cell cultures (Cheung et al, 1988; Bjørge et al, 1994; Hakulinen and Meri, 1994; Junnikkala et al, 1994; Brasoveanu et al, 1995; Mäenpää et al, 1996). In many respects, these systems are markedly different from the actual conditions in solid tumours in vivo (Müller-Klieser, 1987). As the vulnerability of cells to C depends on their interaction with the microenvironment, the properties and functions of C-regulatory mechanisms in three-dimensional systems may differ from those observed in two-dimensional systems.

Tumour cell microspheroids (TMSs) are widely used in in vitro models of tumour nodules (Sutherland, 1988; Carlsson and Nederman, 1992). Like tumour cells in vivo, cells in these spherical aggregates are exposed to gradients of pH, $pO_2$ and nutrients. TMSs are considered to provide a system of compact cells in a cellular microenvironment resembling that found in vivo. In this study, we examined the expression and function of C regulators in ovarian TMSs to develop an in vitro model for analysing the effects of targeted C attack against three-dimensional ovarian tumours.

### MATERIALS AND METHODS

### Antibodies and sera

The rat hybridoma cell line producing the YTH53.1 anti-CD59 monoclonal antibody (MAb) was kindly provided by Professor H Waldmann (Sir William Dunn School of Pathology, University of Oxford, UK). YTH53.1 (IgG2b) was purified from cell culture supernatants using a Protein G Sepharose 4 Fast Flow affinity column (Pharmacia, Uppsala, Sweden). The MAb YTH53.1 was biotinylated by cross-linking biotin to the ε-amino groups via a long arm *N*-hydroxylsuccinimide ester using the biotin labelling method of Vector Laboratories (Burlingame, CA, USA) essentially as described (Jokiranta and Meri, 1993). Lack of activation of the classical C pathway was examined using a C1q binding test (Jokiranta and Meri, 1993). The mouse MAb BRIC230 (IgG1) (anti-DAF) was a generous gift from the International Blood Group Reference Laboratory (Bristol, UK). The mouse MAbs BRIC229 (IgG2b) (anti-CD59), J4-48 (IgG1) (anti-MCP) and aE11 (IgG2a) (anti-C5b-9) were purchased from Bioproducts Laboratories (Elstree, UK), Serotec (Oxford, UK) and Dakopatts (Glostrup, Denmark) respectively. A fluorescein isothiocyanate (FITC)-conjugated goat anti-C3c MAb was purchased from Nordic Immunological Laboratories (Tilburg, The Netherlands). The polyclonal antibody S2 was raised by immunizing a rabbit three times intramuscularly with $10^7$ heat-killed MCF7 cells (breast adenocarcinoma cells) (Hakulinen and Meri, 1994). When appropriate, IgG fractions were prepared by protein G affinity chromatography (Pharmacia). Normal human serum (NHS) was separated from coagulated blood samples from healthy laboratory personnel, divided into 2-ml aliquots and stored at –70°C until used.

### Cell culture

The ovarian teratocarcinoma cell line PA-1 was obtained from American Type Culture Collection (ATCC, Rockville, MD, USA). The cells were grown in monolayers at 37°C in a humidified atmosphere of 5% carbon dioxide in Dulbecco's modified Eagle medium (DMEM; Gibco, Paisley, UK), supplemented with 10% heat-inactivated fetal calf serum (FCS; Gibco), 2 mM L-glutamine (Nord Cell, Bromma, Sweden) and antibiotics [10 units $ml^{-1}$ of penicillin (AL, Oslo, Norway) and 100 μg $ml^{-1}$ of streptomycin (Glaxo, UK)]. Cells were grown in cell culture flasks (Costar, MA, USA) or on coverslips in chambers. Cells were detached from the culture flasks with Versene/EDTA (Gibco), washed twice and resuspended in phosphate-buffered saline (PBS) containing 1% bovine serum albumin (BSA; Sigma).

### Tumour microspheroids (TMSs)

TMSs of PA-1 cells were initiated by seeding cells on 24-well plates (Costar) coated with a thin layer of agarose [0.5% Agarose M (Pharmacia) in distilled water]. PA-1 cells placed into growth medium attached to one another and formed spherical cell aggregates. Cell viability was regularly assessed by microscopic examination after incubation with propidium iodide (Sigma). PA-1 spheroids were grown for an average of 2 weeks and the spheroids with diameters between 100–150 μm were selected for the experiments. Some of the TMSs were harvested, washed and snap frozen in isopentane precooled with liquid nitrogen for immunohistochemical analysis. Fresh TMSs were used for the functional studies.

Initially, we also tried to make spheroids of the ovarian carcinoma cell lines SK-OV-3, Caov-3 and SW626 (ATCC), but their ability to form spheroids was poor.

### Flow cytometry analysis (FCM)

PA-1 cells ($0.2 \times 10^6$) were incubated for 1 h on ice with different concentrations of MAbs directed against CD59 (BRIC229), DAF (BRIC230) or MCP (J4–48) or with the rabbit serum S2 diluted in PBS/BSA and 5% heat-inactivated (56°C, 30 min) NHS. The cells were washed twice in PBS/BSA and incubated on ice for 45 min with FITC-conjugated rabbit anti-mouse Ig (Cappel, Organon Teknika, West Chester, PA, USA) or FITC-conjugated swine anti-rabbit Ig (Dakopatts, Glostrup, Denmark) both diluted 1:50 in PBS/BSA. After washing twice in PBS/BSA, the cells were fixed with 1% paraformaldehyde in PBS and kept at 4°C until analysed. Controls included cells incubated without an idiotype-specific mouse MAb against human B-cell receptor with reactivity with the target cells (provided by Dr Matti Kaartinen at Haartman Institute, Department of Bacteriology and Immunology, University of Helsinki, Helsinki) or normal rabbit serum during the first step. Immunofluorescence was analysed and quantified using a Becton Dickinson FACScan 440 (San Jose, CA, USA) or a Coulter Profile II (Coulter Electronics, Luton, UK) flow cytometer with standard filter set ups.

### Immunofluorescence microscopy

Cryostat sections of TMSs (5 μm) on gelatin-coated slides were fixed in acetone at –20°C for 5 min. After fixation, the sections were incubated with the MAbs BRIC229, BRIC230 and J4–48 (1 μg $ml^{-1}$) or with S2 (dilution 1:100) in a moist chamber according to the immunofluorescence labelling protocol described above. After the final washes in PBS/BSA, the sections were mounted in Mowiol (Heimer and Taylor, 1974). A Leitz microscope (Heidelberg, Germany) equipped with standard fluorescein optics was used for the immunofluorescence analysis.

PA-1 cells grown on coverslips were stained similarly, except that in the standard assay the specimens were not fixed in acetone before the staining procedure.

### Penetration of antibodies into TMSs

TMSs on agarose-coated plates were incubated with 10 μg $ml^{-1}$ of BRIC229 or heat-inactivated S2 (dilution 1:50) in growth medium for 18 h at 37°C in a humidified atmosphere with 5% carbon dioxide. A mouse monoclonal antibody with no known reactivity with the PA-1 cells and normal rabbit serum were used as controls. After washing (2 × 30 min, 37°C) in PBS, the TMSs were incubated for another 18 h with the FITC-conjugated $F(ab')_2$ rabbit anti-mouse Ig (Dakopatts) or FITC-conjugated $F(ab')_2$ donkey anti-rabbit Ig (Jackson Immunoresearch Laboratories, PA, USA).

 © Cancer Research Campaign 1997

**Figure 1** Immunofluorescence examination of complement regulator expression on cultured PA-1 cells (**A–D**) and cryostat sections of PA-1 cells grown in tumour microspheroids (**E–H**). Specimens were stained for MCP (**A** and **E**), DAF (**B** and **F**) and CD59 (**C** and **G**) with the MAbs J4–48, BRIC230 and BRIC229 respectively. Controls were stained with an unrelated mouse MAb (**D** and **H**). **A–D**, ×300; **E–H**, ×40

© Cancer Research Campaign 1997

**Figure 2** Flow cytometric analysis of MCP, DAF and CD59 on PA-1 cells in suspension. The cells were labelled with anti-MCP, anti-DAF or anti-CD59 MAb or with a control MAb (curve on the left in each panel) followed by FITC-conjugated rabbit anti-mouse Ig

After washing, the TMSs were fixed in 1% paraformaldehyde for 2 h and analysed on a BioRad MRC 1000 confocal laser scanning microscope, CLSM (Bio-Test, Rygge, Norway) with a crypton–argon laser. TMSs were scanned in a plane 50–100 µm into the spheroid using the 488 nm excitation line. Alternatively, the fixed TMSs were snap frozen, sectioned and examined in a Leitz immunofluorescence microscope.

### Deposition of C3 and C5b-9 on PA-1 cells after C activation

PA-1 cells ($2 \times 10^5$) in suspension were incubated for 1 h on ice in 10% heat-inactivated S2 diluted in PBS/BSA. After washing twice in Veronal-buffered saline, pH 7.4 (VBS), the PA-1 cells were further incubated with 100 µl of 10% NHS diluted in VBS for 30 min at 37°C. In controls, the classical and alternative C pathways were blocked by adding 0.01 M EDTA to the reaction mixture. After washing three times in PBS/BSA, the cells were immunostained with the anti-C5b-9 MAb aE11 (2 µg $ml^{-1}$) or with the FITC-conjugated goat anti-human C3c (diluted 1:40) and processed for FCM as described above. Negative controls included unsensitized cells incubated with NHS. TMSs were examined in a similar fashion, except that the TMSs were incubated for 18 h with S2 during the sensitization step and that C depositions were analysed by immunofluorescence staining of cryostat sections of TMSs.

### C-mediated lysis of PA-1 cells

Washed TMSs grown for 2 weeks were labelled with 100 µCi $^{51}$chromium ($^{51}Cr$; $Na^{51}CrO^4$; Amersham, UK) (approximately 500 TMSs in 1 ml of DMEM) for 12 h at 37°C, with occasional shaking. This incubation time has earlier been shown to lead to efficient uptake of $^{51}Cr$ into the TMSs (Jääskeläinen et al, 1989). To remove unbound $^{51}Cr$, the TMSs were washed twice in DMEM and incubated for a further 30 min at 37°C in 500 µl of DMEM, before repeating the washing procedure. During labelling the TMSs were incubated simultaneously with appropriate dilutions of antibodies (S2 and/or biotinylated YTH53.1). Final concentrations of reagents (given in 50 µl of DMEM) are indicated in the figure legends. In controls, the individual effects of each of the reagents were examined by replacing them with equivalent amounts of DMEM. TMSs (approximately 20–30 per tube in 50 µl of DMEM) were then incubated with 25% NHS (in a final volume of 200 µl) for 30 min at 37°C. After centrifugation (500 *g* for 5 min), 100 µl samples of the supernatants were carefully removed and counted in a gammacounter. $^{51}Cr$-release in the absence of antibodies was taken as a background (range: 0.5–1.0% of total label incorporated) and release by 0.1% Nonidet P40 (BDH Laboratories Supplies, Poole, UK) as total release of radioactivity. During a 30-min incubation, 0.1% NP40 released 8% of total $^{51}Cr$ incorporated. Cell lysis in the TMSs was determined as percentage of antibody plus complement-induced $^{51}Cr$ release into the supernatant. Examination of C-mediated lysis of PA-1 cells in suspension was performed in a $^{51}Cr$-release assay as described earlier (Hakulinen and Meri, 1994). A total of $5 \times 10^7$ cells were labelled with 25 µCi of $^{51}Cr$ in 150 µl of DMEM. After washes and a 30-min reincubation to release loosely bound $^{51}Cr$, the cells were aliquoted ($10^5$ cells per tube) for the $^{51}Cr$-release assay, which was performed in a similar way to that described for TMSs above.

### RESULTS

### Spheroid formation and growth

Aggregates of PA-1 cells were observed as early as 2–3 days after seeding on agar gels. Large spheroids of densely packed cells with a regular round shape were formed at the end of the first week. The viability of the cells within the spheroids was repeatedly checked by staining with propidium iodide, which showed that only a few nuclei, if any, outside a central core adsorbed the dye.

### Expression of complement regulatory proteins on PA-1 cells and spheroids

PA-1 cells grown on coverslips showed a membranous staining for both MCP and CD59 with the J4–48 and BRIC229 antibodies respectively (Figure 1A and C). No reactivity was seen with the anti-DAF MAb BRIC230 or the control mouse MAb (Figure 1B and D).

© Cancer Research Campaign 1997

**Figure 3** Confocal microscopy analysis of binding of antibodies to the TMSs. TMSs were cultured on agar-coated plates in the presence of the anti-CD59 MAb BRIC229 (**A**) or the polyclonal rabbit antibody S2 (**C**) (18 h, 37°C) followed by FITC-conjugated rabbit anti-mouse Ig or FITC-conjugated donkey anti-rabbit Ig (18 h, 37°C). TMSs were fixed in paraformaldehyde (1%, 2 h) and analysed by confocal laser scanning microscopy. Controls were cultured with an unrelated mouse MAb (**B**) or normal rabbit serum (**D**) in the first step. BRIC229 bound to the outermost 4 or 5 cell layers (**A**), whereas S2 bound very strongly to the outer 7 or 8 cell layers, more weakly to the next 2 or 3 cell layers and not to the most centrally located cells (**C**)

FCM was used to further analyse the expression of complement regulatory proteins on the surface PA-1 cells in suspension. These experiments showed that CD59 and MCP were expressed on the surface of the cells. The widths of the unimodal fluorescence profiles for both molecules were narrow, and more than 90% of the cells were stained. The cells were not, or only very weakly, stained with the anti-DAF MAb BRIC230 (Figure 2).

Sections of TMSs showed strong staining for both MCP and CD59, with a homogeneous pattern throughout the TMSs. All cells showed a prominent circumferential linear membrane staining (Figure 1E and G). The staining pattern for DAF was different. Distinct foci in the outer rim stained positive with the BRIC230 (Figure 1F). The fluorescence was located to the surface membrane of the cells. TMSs incubated with the control MAb during the first incubation step were not stained (Figure 1H).

### Binding of the polyclonal S2 antibody to PA-1 cells and spheroids

PA-1 cells grown on coverslips showed a strong membranous staining for the polyclonal S2 antibody. FCM confirmed this and showed a strong reactivity of S2 with the cells. The width of the unimodal fluorescence profile was broad, and 99.1% of the cells were positive for S2 IgG with a mean fluorescence index of 629.7 (background 10.9). Staining of sections of TMSs with the S2 antibody also showed a strong fluorescence. The staining pattern was homogeneous, and all cells showed a prominent circumferential linear membrane staining. Cells incubated with the preimmune rabbit serum during the first incubation step showed no staining (data not shown).

### Access of antibodies to PA-1 cells in the spheroids

Incubation of fresh TMSs for 18 h with the BRIC229 anti-CD59 MAb only stained the outermost 4 or 5 cell layers when analysed by the confocal laser scanning microscopy. The antibody gave a clear and distinct surface membrane staining. The more centrally located cell layers showed no staining. There was no transition zone between the positively and negatively stained areas (Figure 3A). After exposure of the TMSs to S2 as many as 7 or 8 outer cell layers showed a strong positive staining. The next 2 or 3 more centrally located cell layers showed a weaker staining, thereby constituting a transition zone. The most centrally located cells were not stained (Figure 3C). These findings were confirmed by immunofluorescence microscopy of cryostat sections of TMSs subjected to the same penetration experiments (data not shown). Control TMSs incubated with an irrelevant MAb or normal rabbit serum during the first incubation step showed no (Figure 3B) or weak, non-specific (Figure 3D) staining.

© Cancer Research Campaign 1997

**Figure 4** Flow cytometric and scatter profile analysis of antibody-induced deposition of C3 (**A** and **C**) and C5b-9 (**D** and **F**) on PA-1 cells in suspension. The cells were sensitized with the polyclonal rabbit antibody S2 and incubated with 10% NHS (30 min, 37°C) in the presence (■; **B** and **E**) or absence (□; **C** and **F**) of 0.01 M EDTA. Cells were stained with FITC-conjugated goat anti-C3c Ab (A–C) or with the aE11 anti-C5b-9 MAb followed by FITC-conjugated rabbit anti-mouse Ig (D–F). C3 and C5b-9 were detected on 95% and 60% of the PA-1 cells in suspension respectively

**Figure 5** Immunofluorescence analysis of antibody-induced deposition of C3 and C5b-9 on PA-1 TMSs. TMSs were sensitized with the polyclonal rabbit antibody S2 and incubated with 10% NHS (30 min, 37°C) in the absence (**A** and **C**) or presence (**B** and **D**) of 0.01 M EDTA. Cryostat sections of TMSs were stained with FITC-conjugated anti-C3c (**A** and **B**) or with the aE11 anti-C5b-9 MAb followed by FITC-conjugated rabbit anti-mouse Ig (**C** and **D**). Note deposition of both C3 and C5b-9 on the outer cell layers of the TMSs

 © Cancer Research Campaign 1997

**Figure 6** Complement-mediated killing of PA-1 cells in suspension (**A**) or TMSs (**B**) in the presence (●) or absence (○) of biotinylated YTH53.1 (anti-CD59). $^{51}$Cr-labelled cells and TMSs (50 µl) were sensitized with different amounts of the polyclonal rabbit antibody S2 (50 µl) in the presence or absence of B-YTH53.1 (25 µg ml$^{-1}$) and exposed to NHS (25%) in final volume of 200 µl (37°C, 30 min). Cell lysis (mean ± s.d.) was quantified as release of $^{51}$Cr into the medium. Significantly, more PA-1 cells in suspension were killed in the presence than absence of B-YTH53.1 (*$P < 0.05$). Under similar conditions, the PA-1 cells in spheroids were resistant to C lysis

### C3 and C5b-9 on PA-1 cells and spheroids after C activation

After activation of the classical pathway of C (10% serum) by S2, C3 and C5b-9 were detected on more than 95% and 60% of the PA-1 cells in suspension respectively (Figure 4). None of the tested tumour antigen-reactive mouse MAbs (Bjørge et al, 1996) activated C on the surface of PA-1 cells.

To see if C components could penetrate into spheroids, fresh TMSs were sensitized with S2 for 18 h and thereafter treated with NHS (30 min, 37°C). Cryostat sections of the spheroids stained positive for both C3c and C5b-9. The staining patterns were similar and, interestingly, only the outer 2–5 layers were stained (Figure 5). No binding of the anti-C3c or anti-C5b-9 MAb was seen to cells in suspension or to TMSs that were not sensitized with the S2 antibody. The presence of EDTA in the incubation mixture also prevented C3c and C5b-9 deposition on S2-sensitized cells and spheroids.

### Sensitivity of PA-1 cells and spheroids to C-mediated cytotoxicity

The cytotoxic effect of human C and the functional significance of CD59 in protecting PA-1 cells against C-mediated damage was examined using cells in suspension and in TMSs. PA-1 cells in suspension sensitized with the polyclonal rabbit antibody S2 showed a dose-dependent vulnerability to C-mediated lysis (Figure 6A). Using a saturating concentration of S2, approximately 65% of the cells were killed in the presence of 25% NHS during a 30-min incubation at 37°C. Ten per cent NHS resulted in the killing of 17% of target cells. In the presence of 25% NHS and 25 µg ml$^{-1}$ of the biotinylated anti-CD59 YTH53.1, an average of 88% of PA-1 cells were killed. In comparison, PA-1 cells in TMSs were found to be resistant to C-mediated cytotoxicity (Figure 6B). After sensitization with S2, in the presence of 25% NHS for 30 min, approximately 4% of the cells in the TMSs were lysed, while 8% of the cells in the spheroids were lysed when the biotinylated YTH53.1 MAb was added to the reaction mixture (release of $^{51}$Cr by 0.1% NP40 was taken as total lysis). In the absence of the sensitizing rabbit antibody, no lysis occurred in the two cell systems with the combination of biotinylated YTH53.1 MAb and NHS.

### DISCUSSION

The experiments described in this paper demonstrate that cells in suspension and cells growing in multicellular spheroids differ in their ability to resist C-mediated cytotoxicity. Cells growing in TMSs were much more difficult to kill by C than cells in suspension. This phenomenon was at least partly dependent on differences in the binding of C-activating antibodies to cells in the two systems and possibly on acquisition of an increase in C resistance when the tumour cells are grown in TMSs.

In line with our recent study, we found MCP and CD59 expression, but no or only negligible DAF expression, on PA-1 cells in suspension and on cells growing in monolayers (Bjørge et al, 1996). Similarly, PA-1 cells growing in TMSs also expressed MCP and CD59, and the intensity of expression was homogeneous throughout the TMSs. Interestingly, DAF was also expressed on the PA-1 TMSs. The location of DAF-expressing cells was rather distinct, as only foci in the outer layers in the TMSs expressed DAF. Differences between spheroids and monolayers in the expression of integrins (Brackman, 1995), extracellular matrix components (Brackman, 1995), growth factors (Ness et al, 1994) and growth factor receptors (Ness et al, 1994) have been observed earlier. A possible explanation for these differences is the influence of the distinct cellular microenvironment present in the spheroids (Sutherland, 1988; Carlsson and Nederman, 1992). PA-1 is a

© Cancer Research Campaign 1997

teratocarcinoma cell line that can differentiate into different germ cell layers when growing on non-adhesive surfaces (Zeuthen et al, 1980). On their surface, the cell aggregates contain endoderm-like cells that surround the inner cell mass. As DAF is a marker of early differentiation (Holmes et al, 1992), the induction of its expression could alternatively be explained by the differential organization of cells in the TMSs.

Although both S2 and BRIC229 stained sections of TMSs homogeneously, confocal microscopy showed that these antibodies had only a limited ability to penetrate into live spheroids. These findings were confirmed by ordinary immunofluorescence microscopy on cryostat sections to exclude experimental caveats inherent in the staining protocol used for the confocal laser scanning microscopy analysis. The S2 rabbit IgG penetrated deeper into the spheroids than BRIC229. As it has been predicted that low-affinity antibodies penetrate into tumours more readily than high-affinity antibodies (Fujimori et al, 1989; Langmuir et al, 1992), the difference between BRIC229 and S2 is probably because BRIC229 is a high-affinity MAb (Fletcher et al, 1992), while the S2 IgG probably contains antibodies with different affinities against different antigens. Lack of antibody penetration into the core of TMSs probably reflects the existence of tight intercellular junctions between the cells (Jain, 1988). In addition, cells in the spheroids may have synthesized a glycocalyx (Riethmüller et al, 1993) or a basement membrane-like structure (Zeuthen et al, 1980) that also restrict the access of antibodies into the deeper layers of the TMSs.

Antibody-mediated C activation on PA-1 cells in suspension induced C deposition on nearly all the cells. A similar C attack on the PA-1 TMSs resulted only in deposition of C factors on the outer cell layers of the aggregates. In cell killing experiments, the PA-1 cells were sensitized with the S2 polyclonal antibody. After screening of many monoclonal antibodies, two were found that bound to the PA-1 cells and activated C (C241 against CA19-9 and Ma552 against MUC-1). Unfortunately, the binding of these antibodies to PA-1 cells was too weak to lead to efficient C activation. S2-sensitized PA-1 cells in suspension were shown to be C treated, as almost 65% of the cells were killed after C activation. Neutralization of CD59 enhanced cell killing to more than 85%. In contrast, PA-1 cells growing in TMSs were C resistant; less than 10% of the cells were killed by C. Blocking of CD59 with biotinylated YTH53.1 reduced the resistance of the TMSs to C-mediated lysis only very moderately. The spheroids remained resistant even when higher concentrations of both biotinylated YTH53.1 and NHS were used (data not shown). An earlier study by Buckman et al (1982) obtained similar results by showing that a monoclonal antibody, Fib-75, activated complement on epithelial tumour cells and killed the cells when they were in suspension but not when they were in large clumps.

The difference in PA-1 sensitivity to C lysis between the two systems could be partly determined by the variable access of the C-activating antibodies to the tumour cells. Spheroid tissue may prevent deeper penetration of C until the most superficial cell layers have become damaged, and even after cell damage a barrier of some degree may remain. When high-affinity antibodies are used they may bind avidly to the outermost surface and do not necessarily penetrate deeper into the TMSs. Despite the fact that antibodies bound to TMSs and activated C, no significant C lysis was observed. Recently, we noticed that DAF expression was inversely correlated with the ovarian tumour cells' vulnerability to C-mediated killing (Bjørge et al, 1996). Induced expression of DAF on cells localized at the surface of the TMSs, could also partly explain the increased C resistance of the cells. Other mechanisms, such as rapid depletion of C activity or induced resistance to C attack (Morgan, 1989; Reiter et al, 1992), may also contribute. Further studies are required to see if killing of tumour cells in spheroids can be enhanced by prolonged or repeated C treatment or by employing the antibody-dependent cellular cytotoxicity (ADCC) effector mechanism to the tumour cell killing. It is anticipated that under in vivo conditions ADCC will play an important role in immune attack against tumour cells.

The approach of using MAbs and the C system as an effector to kill tumour cells may be an attractive adjuvant immunotherapeutic alternative (Chapman et al, 1992; Riethmüller et al, 1993). Attempts to use unconjugated C-activating MAbs in the therapy of solid tumours have therefore been tried (Houghton et al, 1985; Goodman et al, 1990; Riethmüller et al, 1993; Rubin, 1993). Although the MAbs bind to the tumour cells in vivo (Houghton et al, 1985; Rubin, 1993) and mediate local C deposition (Houghton et al, 1985), the therapeutic efficiency is limited (Houghton et al, 1985; Riethmüller and Johnson, 1992; Riethmüller et al, 1993; Rubin, 1993) and correlates negatively with the volume of the tumour mass (Riethmüller et al, 1993). Barriers for free access of MAbs and C to the tumour cells and the existence of C resistance mechanisms may limit the success rate (Riethmüller et al, 1993). However, in vitro tests have shown that it is possible to kill tumour cells in suspension using appropriate MAbs and C in combination with targeted neutralization of membrane regulators of complement (Cheung et al, 1988; Bjørge et al, 1994; Hakulinen and Meri, 1994; Junnikkala et al, 1994; Brasoveanu et al, 1995; Mäenpää et al, 1996). The in vivo protocols for this type of immunotherapeutic analysis therefore have to be refined for effective destruction of tumour cells in three-dimensional structures.

Ovarian cancer represents a good candidate for MAb tumour therapy (Rubin, 1993). MAbs can be applied by both the intraperitoneal and intravenous route. If the limitations of MAb penetration and resistance to C can be overcome, the clinical use of MAbs in the management of ovarian cancer could enter a new era.

### ABBREVIATIONS

MAC, membrane attack complex; NHS, normal human serum; TMS, tumour microspheroid

### ACKNOWLEDGEMENTS

This study was supported by grants from The Norwegian Cancer Society, Nordic Academy for Advanced Studies Abroad, Norske Hoechst A/S, BioTest A/S, the Academy of Finland, the Sigrid Jusélius Foundation and the University of Helsinki. Line Bjørge is a recipient of a research fellowship from The Norwegian Cancer Society.

### REFERENCES

Bjørge L, Vedeler CA, Ulvestad E and Matre R (1994) Expression and function of CD59 on colonic adenocarcinoma cells. *Eur J Immunol* **24**: 1597–1603

Bjørge L, Hakulinen J, Wahlstrom T, Matre R and Meri S (1996) Complement regulatory proteins in ovarian malignancies. *Int J Cancer* **68**: 1–12

Brackman D (1995) Effects of 1,25-dihydroxyvitamin D3 on C3H/10T1/2 fibroblasts grown as multicellular spheroids. *Int J Cancer* **62**: 428–435

 © Cancer Research Campaign 1997

Brasoveanu LI, Altomonte M, Gloghini A, Fonsatti E, Coral S, Gasparollo A, Montagner R, Cattarossi I, Simonelli C, Cattelan A, Attadia V, Carbone A and Maio M (1995) Expression of protectin (CD59) in human melanoma and its functional role in cell- and complement-mediated cytotoxicity. *Int J Cancer* **61**: 548–556

Buckman R, McIlnney RA, Shepherd V, Patel S, Coombes RC and Neville AM (1982) Elimination of carcinoma cells from human bone marrow. *Lancet* **2**: 1428–1430

Carlsson J and Nederman T (1992) Tumour spheroids as a model in studies of drug effects. In *Spheroid Culture in Cancer Research*, Bjerkvig R (ed.), pp. 199–216. CRC Press: London

Chapman PB, Scheinberg DA, Dimaggio JJ and Houghton AN (1992) Unconjugated monoclonal antibodies as cancer agents. *Immunol Allergy Clin North Am* **11**: 257–275

Cheung N-KV, Walter EI, Smith-Mensah WH, Ratnoff WD, Tykocinsky ML and Medof ME (1988) Decay accelerating factor protects human tumour cells from complement-mediated cytotoxicity in vitro. *J Clin Invest* **81**: 1122–1128

Fearon DT (1979) Regulation of the amplification C3 convertase of human complement by an inhibitory protein isolated from human erythrocyte membrane. *Proc Natl Acad Sci USA* **76**: 5867–5871

Fletcher A, Bryant JA, Gardner B, Judson PA, Spring FA, Parsons SF, Mallinson G and Anstee DJ (1992) New monoclonal antibodies in CD59: use for the analysis of peripheral blood cells from paroxysmal nocturnal haemoglobinuria (PNH) patients and for the quantitation of CD59 on normal and decay accelerating factor (DAF)-deficient erythrocytes. *Immunology* **75**: 507–512

Fletcher CM, Harrison RA, Lachmann PJ and Neuhaus D (1993) Sequence-specific $^{1}$H-NMR assignments and folding topology of human CD59. *Protein Sci* **2**: 2015–2027

Fujimori K, Covell DG, Fletcher JE and Weinstein JN (1989) Modeling analysis of the global and microscopic distribution of immunoglobulin G, F(ab′)2 and Fab in tumours. *Cancer Res* **49**: 5656–5663

Goodman GE, Hellstrom I, Brodzinsky L, Nicaise C, Kulander B, Hummel D and Hellstrom KE (1990) Phase I trial of murine monoclonal antibody L6 in breast, colon, ovarian, and lung cancer. *J Clin Oncol* **8**: 1083–1092

Guadagni F, Roselli M, Nieroda C, Dansky-Ullmann G, Schlom J and Greiner JW (1993) Biological response modifiers as adjuvants in monoclonal antibody-based treatment (review). *In Vivo* **7**: 591–599

Hakulinen J and Meri S (1994) Expression and function of the complement membrane attack complex inhibitor protectin (CD59) on human breast cancer cells. *Lab Invest* **71**: 820–827

Hamilton TC, Ozols RF and Longo DL (1987) Biologic therapy for the treatment of malignant common epithelial tumours of the ovary. *Cancer* **60** (suppl.): 2054–2063

Heimer G and Taylor CED (1974) Improved mountant for immunofluorescence preparations. *J Clin Pathol* **27**: 254–256

Holmes CH, Simpson KL, Okada H, Okada N, Wainwright SD, Purcell DFJ and Houlihan JM (1992) Complement regulatory proteins at the feto-matemal interface during human placental development: distribution of CD59 by comparison with membrane cofactor protein (CD46) and decay accelerating factor (CD55). *Eur J Immunol* **22**: 1579–1585

Houghton AN, Mintzer D, Cordon-Cardo C, Welt S, Fliegel B, Vadhan S, Carswell E, Melamed MR, Oettgen HF and Old LJ (1985) Mouse monoclonal IgG3 antibody detecting GD3 ganglioside: a phase I trial in patients with malignant melanome. *Proc Natl Acad Sci USA* **82**: 1242–1246

Jain RK (1988) Determinants of tumour blood flow: a review. *Cancer Res* **48**: 2641–2658

Jokiranta TS and Meri S (1993) Biotinylation of monoclonal antibodies prevents their ability to activate the classical pathway of complement. *J Immunol* **151**: 2124–2131

Junnikkala S, Hakulinen J and Meri S (1994) Targeted neutralization of the complement membrane attack complex inhibitor CD59 on the surface of human melanoma cells. *Eur J Immunol* **24**: 611–615

Jääskeläinen J, Kalliomäki P, Paetau A and Timonen T (1989) Effect of LAK cells against three-dimensional tumour tissue. *J Immunol* **142**: 1036–1045

Langmuir VK, Mendonca HL and Wood DV (1992) Comparisons between two monoclonal antibodies that bind to the same antigen but have differing affinities: uptake kinetics and $^{125}$I-antibody therapy efficacy in multicell spheroids. *Cancer Res* **52**: 4728–4734

Meri S, Morgan BP, Davies A, Daniels RH, Olavesen MG, Waldmann H and Lachmann PJ (1990) Human protectin (CD59), an 18.000–20.000 MW complement lysis restricting factor, inhibits C5b-8 catalysed insertion of C9 into lipid bilayers. *Immunology* **71**: 1–9

Morgan BP (1989) Complement membrane attack on nucleated cells; resistance, recovery and non-lethal effects. *Biochem J* **264**: 1–14

Morgan BP and Meri S (1994) Membrane proteins that protect against complement lysis. *Springer Semin Immunopath* **15**: 369–396

Müller-Klieser W (1987) Multicellular spheroids. A review on cellular aggregates in cancer research. *J Cancer Res Clin Oncol* **113**: 101–122

Mäenpää A, Junnikkala S, Hakulinen J, Timonen T and Meri S (1996) Expression of complement membrane regulators membrane cofactor protein (CD46), decay accelerating factor (CD55) and protectin (CD59) in human malignant gliomas. *Am J Pathol* **148**: 1139–1152

Ness GO, Pedersen P-H, Bjerkvig R, Laerum OD and Lillehaug JR (1994) Three-dimensional growth of glial cell lines affects growth factor and growth factor receptor mRNA levels. *Exp Cell Res* **214**: 433–436

Nicholson-Weller A, Burge J, Fearon DT, Weller PF and Austen KF (1982) Isolation of a human erythrocyte membrane glycoprotein with decay-accelerating activity for C3 convertases of the complement system. *J Immunol* **129**: 184–189

Pangburn MK, Schreiber RD and Müller-Eberhard HJ (1983) Deficiency of an erythrocyte membrane protein with complement regulatory activity in paroxysmal nocturnal hemoglobinuria. *Proc Natl Acad Sci USA* **80**: 5430–5434

Reiter Y, Ciobotariu A and Fishelson Z (1992) Sublytic complement attack protects tumour cells from lytic doses of antibody and complement. *Eur J Immunol* **22**: 1207–1213

Riethmüller G and Johnson JP (1992) Monoclonal antibodies in the detection and therapy of micrometastatic epithelial cancers. *Curr Opin Immunol* **4**: 647–655

Riethmüller G, Schneider-Gädicke E and Johnson JP (1993) Monoclonal antibodies in cancer therapy. *Curr Opin Immunol* **5**: 732–739

Rollins SA and Sims PJ (1990) The complement inhibitory activity of CD59 resides in its capacity to block incorporation of C9 into membrane C5b-9. *J Immunol* **144**: 3478–3485

Rubin SC (1993) Monoclonal antibodies in the management of ovarian cancer. A clinical perspective. *Cancer Suppl* **71**: 1602–1612

Schönermark S, Rauterberg EW, Shin ML, Loke S, Roelcke D and Hänsch GM (1986) Homologous species restriction in lysis of human erythrocytes: a membrane-derived protein with C8-binding capacity functions as an inhibitor. *J Immunol* **136**: 772

Seya T, Turner J and Atkinson JP (1986) Purification and characterization of a membrane protein (gp45–70) which is a cofactor for cleavage of C3b and C4b. *J Exp Med* **163**: 837–855

Stefanová I, Hilgert I, Kristová H, Brown R, Low MG and Horejsi V (1989) Characterization of a broadly expressed human leucocyte surface antigen MEM-43 anchored in the membrane through phosphatidylinositol. *Mol Immunol* **26**: 153–161

Sugita Y, Nakano Y and Tomita M (1988) Isolation from human erythrocytes of a new membrane protein which inhibits the formation of complement transmembrane channels. *J Biochem* **104**: 633

Sutherland RM (1988) Cell and environment interactions in tumour microregions: the multicell spheroid model. *Science* **240**: 177–184

Vogel C-W (1991) Complement, a biologic effector mechanism for tumour cell killing. *Immunol Allergy Clin North Am* **11**: 277–299

Zalman LS, Wood LM and Müller-Eberhard HJ (1986) Isolation of a human erythrocyte membrane protein capable of inhibiting expression of homologous complement transmembrane channels. *Proc Natl Acad Sci USA* **83**: 6975

Zeuthen J, Nørgaard JOR, Avner P, Fellous M, Wartiovaara J, Vaheri A, Rosen A and Giovanella BC (1980) Characterization of a human ovarian teratocarcinoma-derived cell line. *Int J Cancer* **25**: 19–32

© Cancer Research Campaign 1997